# Periduodenal Abscess

**Published:** 1903-04-04

**Authors:** 


### Hospital Clinics and Medical Progress.

### PERIDUODENAL ABSCESS.

In an address delivered before the Surgical Section of the New York Academy of Medicine and reported in the *New York Medical News*, Dr. Bainbridge took for his subject periduodenal abscess secondary to ulcer of the duodenum. His remarks are based mainly on an exhaustive investigation of recorded cases, together with personal observations of the affection. The difficulty in correctly diagnosing perforation of the duodenum is shown by the fact that out of 51 recorded cases in only three was the condition recognised previous to operation or autopsy, while 18 out of the 51 presented a perfect clinical picture of perforative appendicitis, and operation was performed under the belief that the primary lesion would be found in this situation. We must frankly admit, he says, that with our present knowledge, in the vast majority of cases, the diagnosis must be general peritonitis with the site of perforation undecided. Scant information can be obtained from medical literature on the form of perforative ulcer of the duodenum which does not involve the general cavity of the peritoneum. Out of 179 cases of subphrenic abscess collected by Maydl six were due to perforating ulcer of the duodenum. There are 26 cases of periduodenal abscess due to perforating ulcer of the duodenum recorded in all; 22 of these ended fatally without the diagnosis being made. In two the condition was only discovered at the time of operation, and one of these recovered. In only two was the site of the lesion recognised previously to operation or post-mortem examination, neither of these patients recovered, the mortality for the 26 cases thus working out at a little over 96 per cent. Dr. Bainbridge reports the following illustrative case from his own practice. The patient was 54 years of age, and there was nothing of importance to be noted in his previous history except an attack of appendicitis 13 months before, which lasted seven days. From that time on he seemed in good health, except that he occasionally noticed slight tenderness in the right iliac region. When taken ill on June 20th, 1899, the patient appeared to be suffering from acute gastritis. The illness commenced suddenly with severe vomiting and intense abdominal pain. Some hours after the commencement of the attack pain and tenderness were still marked over the region of the stomach. Rigidity of the abdominal wall was present and was more marked above the level of the umbilicus than below, and the severe symptoms were chiefly localised in the epigastric and right hypochondriac regions. There was constant vomiting, with normal temperature, and pulse 84 per minute. Under medical treatment, which consisted of milk diet, the use of bismuth and the employment of digestives, all symptoms gradually disappeared. At the end of six days the patient was walking about apparently well. Vomiting, however, recommenced, accompanied by epigastric discomfort and some abdominal rigidity. Subsequently the patient steadily emaciated, and died at the end of August, in the eleventh week from the onset of the symptoms. At the autopsy an ulcer, a quarter of an inch in diameter, was found on the posterior wall of the duodenum an inch and a quarter from the pylorus. The ulcer communicated with a large abscess cavity containing about a quart of fetid pus. The abscess involved the head of the pancreas, and was entirely post peritoneal. The remains of old appendicitis were found. Dr. Bainbridge describes other illustrative cases and then turns to a general discussion of the affection. With regard to symptoms, the most frequent are vomiting, pain, tenderness and muscular rigidity in the epigastric and right hypochondriac regions, constipation, sudden onset and shock. Usually there is a history of previous gastric or intestinal disturbance, and perhaps of hæmatemesis or melæna. Complications which are mentioned as having occurred are general peritonitis, pleurisy, empyema, pyopneumothorax, ulcer of the stomach, pancreatitis, and perforation of the diaphragm and lung. The diagnosis is always difficult; peritonitis, with the site of the primary lesion unknown, or appendicitis being the usual diagnoses made in these cases. Treatment there can be no doubt about; early operation as soon as the diagnosis is made, if the patient is in a condition to withstand a laparotomy.

